# Whole genome analysis of host-associated *lactobacillus salivarius* and the effects on hepatic antioxidant enzymes and gut microorganisms of *Sinocyclocheilus grahami*

**DOI:** 10.3389/fmicb.2022.1014970

**Published:** 2022-10-27

**Authors:** Wei-Gang Xin, Xin-Dong Li, Yi-Cen Lin, Yu-Hang Jiang, Mei-Yu Xu, Qi-Lin Zhang, Feng Wang, Lian-Bing Lin

**Affiliations:** ^1^Faculty of Life Science and Technology, Kunming University of Science and Technology, Yunnan Kunming, China; ^2^Engineering Research Center for Replacement Technology of Feed Antibiotics of Yunnan College, Yunnan, Kunming, China

**Keywords:** *Sinocyclocheilus grahami*, *lactobacillus salivarius*, genomic characterization, antioxidant enzymes, gut microbiome

## Abstract

As a fish unique to Yunnan Province in China, *Sinocyclocheilus grahami* hosts abundant potential probiotic resources in its intestinal tract. However, the genomic characteristics of the probiotic potential bacteria in its intestine and their effects on *S. grahami* have not yet been established. In this study, we investigated the functional genomics and host response of a strain, *Lactobacillus salivarius* S01, isolated from the intestine of *S. grahami* (bred in captivity). The results revealed that the total length of the genome was 1,737,623 bp (GC content, 33.09%), comprised of 1895 genes, including 22 rRNA operons and 78 transfer RNA genes. Three clusters of antibacterial substances related genes were identified using antiSMASH and BAGEL4 database predictions. In addition, manual examination confirmed the presence of functional genes related to stress resistance, adhesion, immunity, and other genes responsible for probiotic potential in the genome of *L. salivarius* S01. Subsequently, the probiotic effect of *L. salivarius* S01 was investigated *in vivo* by feeding *S. grahami* a diet with bacterial supplementation. The results showed that potential probiotic supplementation increased the activity of antioxidant enzymes (SOD, CAT, and POD) in the hepar and reduced oxidative damage (MDA). Furthermore, the gut microbial community and diversity of *S. grahami* from different treatment groups were compared using high-throughput sequencing. The diversity index of the gut microbial community in the group supplemented with potential probiotics was higher than that in the control group, indicating that supplementation with potential probiotics increased gut microbial diversity. At the phylum level, the abundance of Proteobacteria decreased with potential probiotic supplementation, while the abundance of Firmicutes, Actinobacteriota, and Bacteroidota increased. At the genus level, there was a decrease in the abundance of the pathogenic bacterium *Aeromonas* and an increase in the abundance of the potential probiotic bacterium *Bifidobacterium*. The results of this study suggest that *L. salivarius* S01 is a promising potential probiotic candidate that provides multiple benefits for the microbiome of *S. grahami*.

## Introduction

*Sinocyclocheilus grahami*, belonging to the family Cyprinidae, subfamily Barbinae, and genus *Sinocyclocheilus*, is unique to Dianchi Lake in Yunnan. Known as one of the “four famous fish,” *S. grahami* is fished, consumed, and considered an important economic fish (Yin et al., 2021). However, because of the destruction of its habitat and invasion by exotic species, *S. grahami* was listed as a Grade II protected animal in 1989, and further as an endangered animal in 1998. In 2007, to save the species from extinction, the artificial reproduction of *S. grahami* was achieved for the first time. After four generations of manual selection and breeding, a high-quality new national variety (“*S. grahami*, Bayou No. 1”) was certified in 2018 (Yin et al., 2021). Nevertheless, during the process of artificial breeding, antibiotics were commonly used to treat diseases in specimens, including gill and skin inflammation (Yang et al., 2007).

In aquaculture, antibiotics are often used as additives to treat and prevent diseases, because they can inhibit the reproduction of bacteria (Vaseeharan and Thaya, 2014). However, antibiotic overuse has led to the emergence of multidrug-resistant pathogens, damaging the environment, and posing a risk to public safety and health. Previous studies have shown that superbugs resistant to multiple antibiotics can be transmitted among animals and humans through contaminated food and water (Davies and Davies 2010). For instance, contamination with *Vibrio parahaemolyticus* was observed in 95 (38.0%) of 250 aquatic product samples from Guangdong, China, among which 90.53% of the strains showed streptomycin resistance (Xie et al., 2017). In January 2020, the Ministry of Agriculture and Rural Affairs of China issued a comprehensive ban on the addition of antibiotics to animal feed to address issues related to drug residues and antibiotic resistance (Zhou et al., 2021). Therefore, the excavation of a safe and effective alternative to antibiotics, including probiotics, is urgently needed.

Many mechanisms have been proposed to explain the positive effects of probiotics, including stimulating the immune system, helping the host to resist the invasion of external harmful substances and disease-curing organisms, and aiding with digestion (Macfarlane and Macfarlane, 1997; Dong et al., 2018; Sun et al., 2021). For instance, Bacillus cereus NY5 can antagonize *Streptococcus lactis* by modulating specific and non-specific immunity in tilapia (Ke et al., 2022). *Lactobacillus rhamnosus* GG normalizes gut dysmotility induced by environmental pollutants (oxytetracycline, arsenic, polychlorinated biphenyls and chlorpyrifos) *via* affecting serotonin level in zebrafish larvae (Wang et al., 2022). *Bifidobacteria* are proved to be capable of relieving colitis symptoms in both *in vivo* and *in vitro* experiments through following potential mechanisms (e.g., enhancing the hosts’ antioxidant activity, decreasing myeloperoxidase activity, and reactive oxygen species, et. al; Yao et al., 2021). Moreover, in contrast with traditional antibiotics, probiotics fight bacterial diseases and treat inflammation without increasing resistance, through their antimicrobial, antioxidant, anti-inflammatory, and immunomodulatory action (Vanderhoof and Young, 1998; Abd El-Ghany et al., 2022; Yang et al., 2022). Also, probiotics used as a water supplement can improve water quality by affecting the microbial communities of the environment and reducing metabolic waste in the water system (Talpur et al., 2013). These characteristics have led to the widespread use of probiotics in animal farming, particularly in aquaculture. In aquaculture, probiotics can regulate and rebuild the microecological balance of the gut, enhance immunity against diverse pathogens, and improve the conversion rate of feed energy and growth performance (Merrifield et al., 2010; Akhter et al., 2015; Hoseinifar et al., 2018; Chauhan and Singh, 2019; Ringø, 2020). For instance, *Lactobacillus salivarius* can inhibit the growth of *Vibrio* spp. within pike-perch larvae, as well as improve ossification and survival rates (Ljubobratovic et al., 2020). However, while different sources of probiotics have shown consistent and favorable results in higher vertebrates, the effects on the gut of fish are variable. Meanwhile, the use of host-associated probiotics as feed additives has a positive effect on fish farming, as reported by Tarkhani et al. (2020). Therefore, probiotic bacteria isolated from the host gut show greater potential to replace antibiotics than non-specific probiotics.

In recent years, increasing evidence has shown that the gut microbiota plays a key role in maintaining health and controlling disease, regulating many important physiological functions of the host (Tran et al., 2018; Tang et al., 2021). Lactic acid bacteria (LAB) and their metabolic derivatives can improve the gut microbiota and enhance the host’s immunity against external harmful substances and pathogenic bacteria (Wang et al., 2021). For instance, feeding crucian carp *Lactococcus lactis* was effective as a treatment for intestinal inflammation and mucosal barrier function damage caused by *Aeromonas hydrophila* (Dong et al., 2018). *Lactobacillus rhamnosus* recovered the growth of zebrafish larvae under perfluorobutanesulfonate exposure *via* its antioxidant properties, reshaping the gut microbiota, and enhancing the production of bile acids (Sun et al., 2021). Previous studies reported that the gut microorganisms and antioxidant system have synergistic effects against harmful external substances in animals (Uchiyama et al., 2022). Interestingly, LAB was reported to be involved in the activation of antioxidizing systems in animals, promoting their adaptation to external changes (Han et al., 2022). However, the effect of LAB on *S. grahami* is not reported. This study is the first to investigate the intestinal microbial changes in *S. grahami* after feeding with host-associated LAB.

*Lactobacillus salivarius*, is a host-associated bacterium previously isolated from the gut of *S. grahami* (Xin et al., 2022). We have isolated and obtained five potential probiotics *with high antibacterial activity* (i.e., two *Bacillus subtilis*, two *Lactobacillus sake* and one *L. salivarius)* from the gut of *S. graham.* Particularly, the bacteriocin *LSP01* produced by *L. salivarius* 01 exhibited the best antibacterial activity against *A. hydrophila*. *L. salivarius* inhibits the growth of pathogens by disrupting cellular activity and inducing pore size formation in *A. hydrophila* cells. Therefore, in this study, *L. salivarius* 01 was used to explore the probiotic potential. The whole genome was sequenced to evaluate the safety and potential properties of the strain at the genetic level. In addition, the effect of *L. salivarius* on the antioxidant capacity of the hepar and intestinal microbial populations of *S. grahami* was assessed using feeding experiments. As a result, *L. salivarius* was confirmed as a safe and effective potential probiotic that can increase the antioxidant capacity and improve the gut microbiota of *S. grahami*.

## Materials and methods

### Bacterial strains and culture conditions

*Lactobacillus salivarius* S01 is a host-associated potential probiotic isolated from the gut of *S. grahami* (bred in captivity) and preserved in Engineering Research Center for Replacement Technology of Feed Antibiotics of Yunnan College (Xin et al., 2022). For routine use, the strains were grown and subcultured in MRS broth at 37°C for 24 h.

### Whole genome sequencing

The total genomic DNA of *L. salivarius* S01 was extracted using the sodium dodecyl sulfate (SDS) method combined with a purification column. Total genomic DNA was sequenced using an ONT PromethION sequencer (Oxford Nanopore Technologies, Oxford, UK). For filtering, low-quality and short-length reads were discarded from the raw reads. The reads were assembled using Unicycler V0.4.9 software (Wick et al., 2017). The annotation of the assembled genome of *L. salivarius* S01 was performed using Prokka V1.12 software (Seemann, 2014). RepeatMasker V4.1.0 software was used to predict repeat sequences in the genome of *L. salivarius* S01. The prediction of pseudogenes of *L. salivarius* S01 was performed using Pseudofinder software. MinCED V0.4.2 software was used to predict the sequence of Clustered Regularly Interspaced Palindromic Repeats (CRISPRs) on the chromosome of *L. salivarius* S01. Genomic islands in the genome of *L. salivarius* S01 were predicted by IslandViewer 4 (http://www.pathogenomics.sfu.ca/islandviewer/). The prophage in the genome of *L. salivarius* S01 was predicted using PhiSpy (https://github.com/linsalrob/PhiSpy).

The predicted gene sequences were compared using several functional databases, including Cluster of Orthologous Groups (COG; Tatusov et al., 2000), Kyoto Encyclopedia of Genes and Genomes (KEGG; Kanehisa et al., 2004), Swiss-Prot, and RefSeq, using BLAST^+^ (2.5.0^+^). The gene function annotation results were obtained, followed by gene function annotation analysis, such as COG and KEGG metabolic pathway enrichment analysis and gene ontology (GO) function enrichment analysis. Finally, the secondary metabolism gene cluster was analyzed using antiSMASH (v5.2.0), while the bacteriocin synthesis gene cluster of the strain was analyzed using BAGEL4 (Blin et al., 2019).

The presence of antimicrobial resistance genes was compared using the comprehensive antibiotic resistance database (CARD) (McArthur et al., 2013). The CARD database is constructed as the Antibiotic Resistance Ontology (ARO) taxonomic unit to correlate information on antibiotic modules and their targets, gene variants, etc.

### Antibiotic sensitivity

Standard disc diffusion was performed for antibiotic susceptibility testing according to the Clinical and Laboratory Standards Institute (CLSI; Keter et al., 2022). *L. salivarius* S01 were cultured on MRS broth for 24 h to determine the antibiotic sensitivity of selected strains to antibiotic (i.e., tetracycline, erythromycin, penicillin, ampicillin, and chloramphenicol). The experiment was repeated three times independently.

### Tolerance To gastrointestinal conditions

An *in vitro* artificially simulated gastrointestinal juices model was applied, following the previously reported methods, with minor modifications (Li et al., 2020). The simulated gastric juice was prepared by adding 10 g/l pepsin (Solarbio, Beijing, China) to 16.4 ml of sterile 0.1 mol/l HCL, filtered through a membrane with 0.22 μm pore, and adjusted to pH 2.0, 3.0, and 4.0 using sterile 1 mol/l NaOH. The simulated intestinal juice was prepared by adding 10.0 g/l trypsin (Solarbio, Beijing, China) and 6.8 g of KH_2_PO_4_ to 500 ml of sterile ddH_2_O. The pH was adjusted to pH 6.8 using 1 mol/l NaOH and filtered through a membrane with 0.22 μm pore. One milliliter of *L. salivarius* S01 (approximately 10^7^–10^8^) suspension was inoculated into 5 ml of simulated gastric juice at pH 2.0, 3.0, and 4.0, and incubated for 3 h at 37°C. One milliliter of *L. salivarius* S01 (approximately 10^7^–10^8^) suspension was also inoculated into 5 ml of simulated intestinal juice for 4 h. Then, the bacterial solutions were cultured on MRS agar for 24 h to determine the tolerance of selected strains to simulated gastrointestinal juice: Survival rate (%) = lg N1 / lg N2 × 100%, where N2 is the total viable counts of the selected strains at 0 h and N1 is the total viable counts after exposure to the simulated gastrointestinal juice for different time periods.

### Fish husbandry and experimental methods

The *S. grahami* specimens used for the experiments were donated by Yiliang jianzhiyuan Food Co., Ltd. (Kunming, Yunnan). After one week of quarantine, a total of 60 healthy, non-injured, and undeformed *S. grahami* fingerlings (8.0 ± 1.5-mm long) were randomly assigned to six continuously aerated 300-L aquariums, equipped with temperature and oxygen supply control devices.

The experiment was comprised of two groups: the control group (n = 3, 10/cylinder), named as LSC, which was fed basal feed (Satura, Kunming, China), and the treatment group (n = 3, 10/cylinder), named as LSM, which was fed the basal diet supplemented with *L. salivarius* (approximately 1 × 10^7^ CFU/g). All samples (60) were treated. *L. salivarius* S01 was inoculated in MRS broth with 1% pitch rate and incubated at 37°C for 24 h. Afterwards, bacterial cultures were centrifuged at 4,000 × g for 10 min, supernatant was discarded, and cell pellet was resuspended in PBS solution. *L. salivarius* S01 was mixed with the diet and homogenized, and pelleted using oscillating granulator (Daxiang, Guangzhou, China) with 125 μm mesh. At last, mixed diet was dried at room temperature in a ventilated room. Dried pellets for plate-coating inspection. *L. salivarius* S01 (approximately 1 × 10^7^ CFU/g) was measured by viable bacteria. The specimens were fed a quantity of 3% of their body weight twice a day for 28 consecutive days. The light–dark cycle ratio was 14 h: 10 h, and all water quality standards, including temperature (18 ± 0.5°C), pH (8.0 ± 0.5), and DO (8.5 ± 0.12 ppm), were monitored daily. Half of the water in the tank was replaced each day to ensure the best growth conditions for the fish. The experimental animals were processed in accordance with the recommendations from the Guide for the Care and Use of Laboratory Animals. The experimental protocol was approved by the Ethics Committee of Research of Kunming University of Science and Technology.

### Sampling for analysis

At the end of the feeding experiment and after fasting for 24 h, three fish were randomly selected from the control and experimental groups, respectively, and the tissue samples were collected. The hepar of *S. grahami* were collected by dissection, then triturated in pre-cooled homogenization medium (0.01 M Tris–HCl, 0.001 M EDTA-Na_2_, and 0.01 M sucrose, pH 7.4). *S. grahami* hepar were collected by autopsy and ground in pre-chilled homogenizing medium (0.01 M Tris–HCl, 0.001 M EDTA-Na_2_, and 0.01 M sucrose, pH 7.4), and centrifuged (4,000 × *g* for 10 min at 4°C). The resulting supernatant was collected and stored at −80°C for subsequent analysis of hepatic antioxidant enzyme activity. Gut samples from the same fish were collected, placed in Eppendorf tubes, and stored at −80°C.

### Hepatic antioxidant enzyme activity assay

The supernatant was incubated with the enzyme-substrate and read at the indicated wavelength using a UV-8000ST spectrophotometer (Shanghai Yuanxi Instruments Co., Ltd.). The enzyme activity assay was performed in triplicate. The catalase (CAT; E.C.1.11.1.6), superoxide dismutase (SOD; E.C.1.15.1.1), and peroxidase (POD; E.C.1.11.1.7) activities and malondialdehyde (MDA) levels were determined. Commercial assay kits were purchased from Nanjing Jiancheng Institute of Bioengineering (Nanjing, China), and all enzyme activity assays were measured, according to the kit instructions. CAT activity was measured using the CAT Activity Assay Kit (cat. no. A007-1-1). CAT can catalyze the decomposition of hydrogen peroxide, and ammonium molybdate can quickly prevent the decomposition of hydrogen peroxide. The remaining hydrogen peroxide can quickly combine with ammonium molybdate to form a pale-yellow complex that can be measured at 405 nm. SOD activity was measured using a total superoxide dismutase (T-SOD) detection kit at 550 nm (hydroxylamine method; cat. no. A001-1-2). The change in POD activity was measured using a peroxidase assay kit at 420 nm (cat. no. A084-1-1). The MDA levels were measured using the Malondialdehyde Assay Kit at 532 nm (TBA method) (cat. no. A003-1-1).

### Gut microbiota analysis

The fish gut samples were sent to Shanghai Majorbio Bio-pharm Technology Co., Ltd. for sequencing. The sequencing primers were primer 338F (ACTCCTACGGGAGGCAGCAG) and primer 806R (GGACTACHVGGGTWTCTAAT) for amplifying the V3-V4 region of 16S rRNA gene. Sequence analysis was performed using QIIME 1.7 and FLASH 1.2. QIIME 1.7 was used to remove low-quality fragments from the original reads, and FLASH 1.2 was used to complete read merging. Operational taxonomic units (OTUs) were clustered using Uparse based on the threshold of the similarity being above 97%. The RDP classifier algorithm was used to compare the 97% similar OTU representative sequences with the SILVA database for taxonomic analysis.

### Statistical analysis

Gut microbial data validated by multiple comparisons (Knight et al., 2018). Student’s *t*-test (two-tailed test) was used to identify significant differences between two groups in phylum and genus level. Multiple testing adjustment of the data by Benjamini-Hochberg (BH).

All experiments were performed in triplicate, and the results were presented as the mean ± standard deviation (SD). Independent samples *t*-test (two-tailed test) was used to evaluate between-group variance. One-way ANOVA plus least significant difference (LSD) method was employed to analyze multi-group significance. *p*-values <0.05 were considered significant.

## Results

### Draft genome sequencing, assembly, and mapping

**Figure 1:**
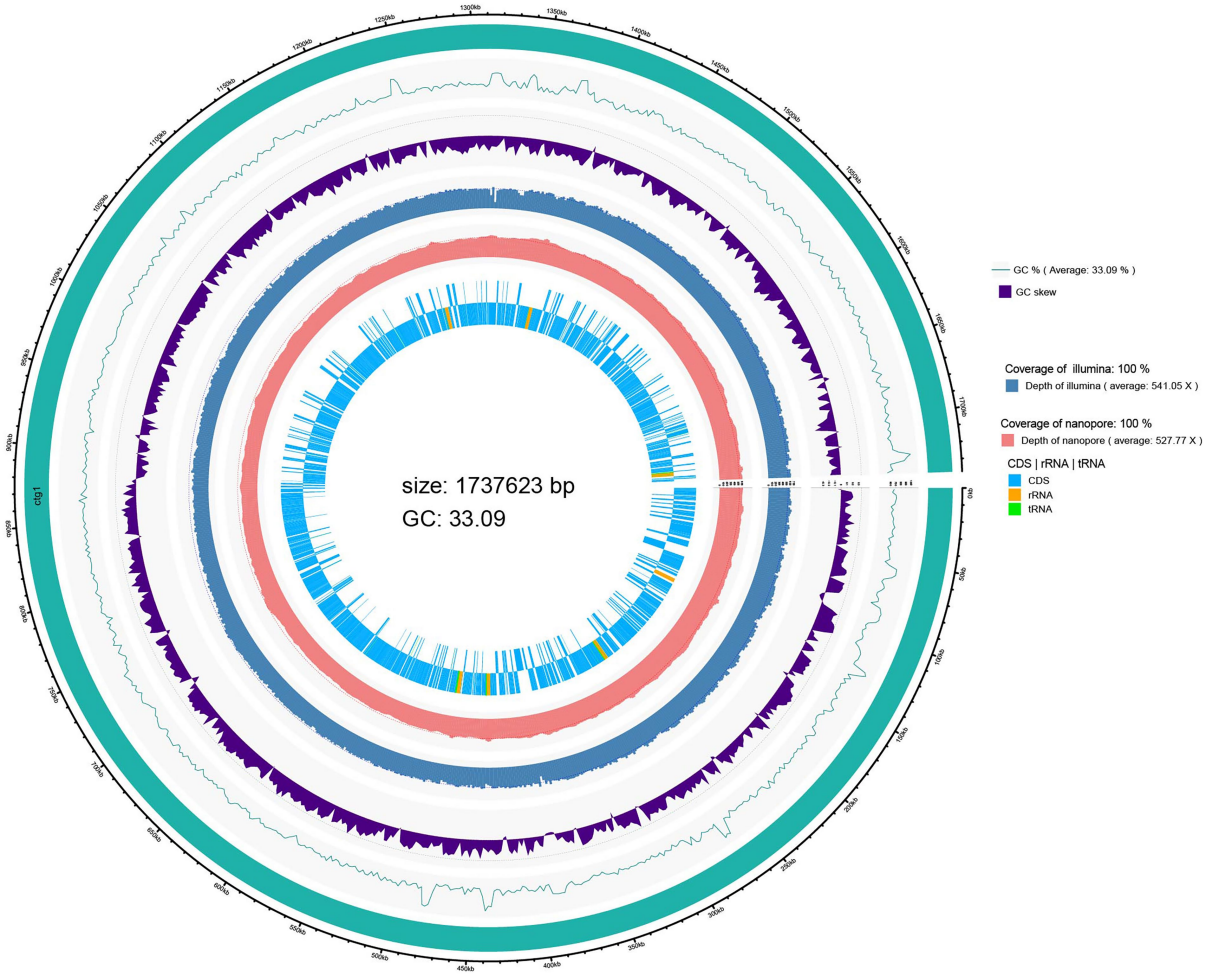
Circular genome map of *L. salivarius* S01.

A genome circle map drawn by integrating the predicted genome information annotation is shown in Figure 1. The innermost circle shows the coding regions (CDS) and non-coding RNA regions (rRNA and tRNA) of the genome. The whole genome sequencing results showed that the genome size of *L. salivarius* S01 was 1,737,623 bp with a GC content of 33.09%. A total of 1753 coding DNA sequences (CDSs), 22 rRNA operons (including 7 23S rRNA, 7 16S rRNA, 8 5S rRNA), and 78 transfer RNA (tRNA) genes were found in the genome of *L. salivarius* S01. In addition, one CRISPR sequence and four gene islands were predicted.

The CDSs on the chromosome of *L. salivarius* S01 were annotated using the COG database, and the results are shown in Supplementary Figure S1. One thousand and five genes were annotated into 21 functional categories through the COG database. The major COGs were translation/ribosomal structure and biogenesis (167), amino acid transport and metabolism (106), carbohydrate transport and metabolism (88), replication/recombination/repair (78), and cell wall/membrane/envelope biogenesis (72). Furthermore, GO analysis revealed that 470 genes were classified into biological processes, 991 genes were classified into cellular components, and 1,037 genes were associated with molecular functions (Figure S1). One thousand and hundred ninety genes involved in KEGG metabolic pathway analysis were classified into five major categories: Metabolism class (866), followed by Genetic Information Processing (183), Environmental Information Processing (112), Organismal Systems (15), and Cellular Processes (14; Supplementary Figure S2).

**Figure 2:**
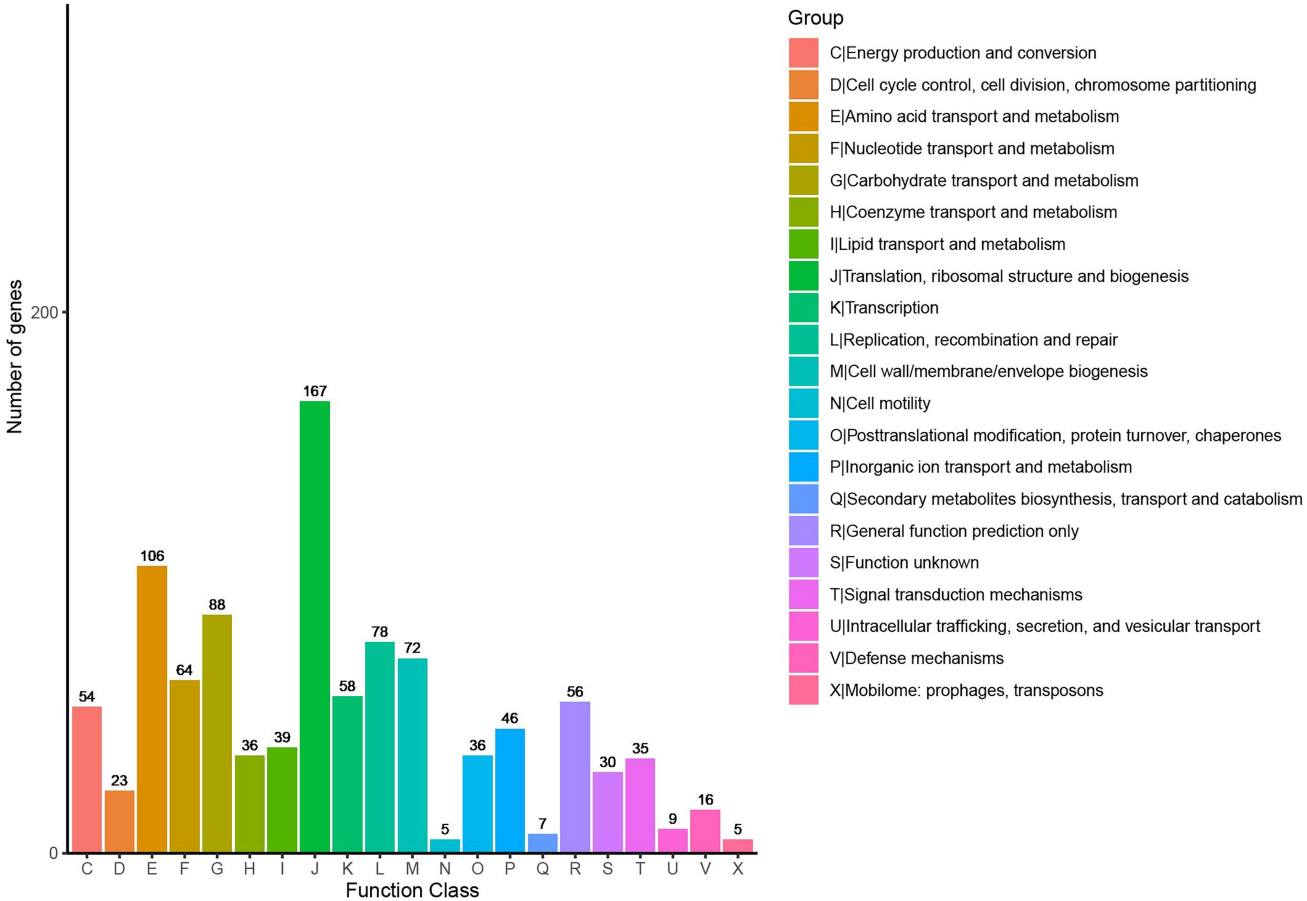
Distribution of genes across COG functional categories in the genome of *L. salivarius* S01.

### Genomic characterization of probiotic traits

Genes for the following probiotic features were examined: tolerance to stress conditions, aid in adhesion and colonization, antioxidative stress immunity, and protective repair of DNA and proteins. The genomic analysis detected 21 genes encoding proteins that may be related to the tolerance of digestive enzymes, bile salts, and acidic environments. Furthermore, genes related to immune response against oxidative stress, and protein and DNA molecular repair protection were also present in the genome (Table 1).

**Table 1:** Probiotic-related genes present in *L. salivarius* S01.

**Gene**	**Putative function**	**Locus tag**
**Stress resistance genes**		
*dna*J	Temperature tolerance	M9Y03_02835
*htp*X	Temperature tolerance	M9Y03_01245
*dna*K	Temperature tolerance	M9Y03_02830
*nha*C_1	Acid resistantce	M9Y03_04420
*nha*C_2		M9Y03_04070
**DNA and protein protection and repair**		
*clp*b	Persistence capacity *in vivo*	M9Y03_04110
*clp*p		M9Y03_05525
*msr*B	Persistence capacity *in vivo*	M9Y03_00160
**Adhesion ability**		
*dna*K	Mucin binding	M9Y03_02830
*gnd*A	Promotes adherence to epithelial cells	M9Y03_03165
*eno*	Collagen binding	M9Y03_05500
**Immunomodulation**		
*dna*K	Protection against osmotic shock	M9Y03_02830
*trxA*	NADPH-depended oxidoreductase activity	M9Y03_06945
*trx*B	NADPH-depended oxidoreductase activity	M9Y03_08055

### Genomic characterization of antibacterial substances production

**Figure 3:**
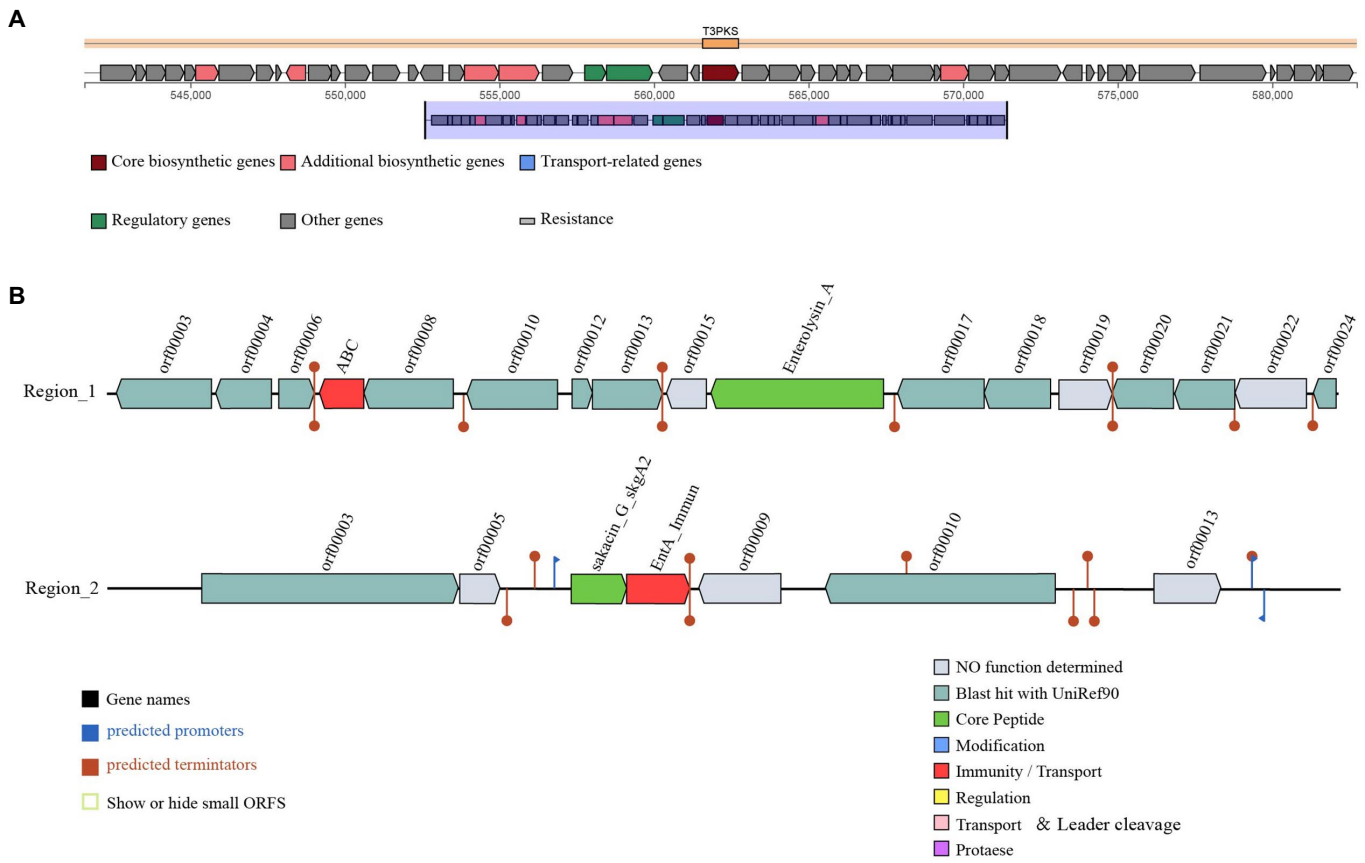
Prediction of antimicrobial-associated protein structures in the genome of *L. salivarius* S01. **(A)** Synthetic gene cluster of polyketide synthase (T3PKS; based on antiSMASH database prediction). **(B)** Two bacteriocin (region_1: Enterolysin_A and region_2: Sakacin_G) synthesis gene clusters (predicted based on the BAGEL4 database).

Three gene clusters related to antibacterial substances synthesis were predicted in the *L. salivarius* S01 genome (Figure 3). The antiSMASH database predicted the existence of a polyketide synthase (T3PKS) synthesis gene cluster in the genome. Based on BAGEL4 platform, the results showed that the genome contained two bacteriocin synthesis gene clusters, as predicted: Enterolysin A as the core gene, including one immune gene and multiple transporter genes, and sakacin_G_skgA1 (class II bacteriocin) as the core gene, including a bacteriocin immune protein gene and a replication initiation protein gene.

### Resistance genotypes and phenotypes

The predicted results of antibiotic resistance genes are shown in Table 2. The identities of *tet (L)* and *ErmC* were 98.03 and 93.85%, respectively; The identities of *Escherichia coli EF-Tu mutants conferring resistance to kirromycin* and *Staphylococcus aureus rpoB mutants conferring resistance to rifampicin* were 73.03 and 72.14%, respectively; The identities of other antibiotic resistance genes was less than 70%.

**Table 2:** Antimicrobial resistance genes present in *L. salivarius* S01.

**Best_Hit_ARO**	**Best_Identities**	**Drug Class**
*tet(L)*	98.03%	tetracycline antibiotic
*ErmC*	93.85%	macrolide antibiotic; lincosamide antibiotic; streptogramin antibiotic
*Escherichia coli EF-Tu mutants conferring resistance to kirromycin*	73.03%	elfamycin antibiotic
*Staphylococcus aureus rpoB mutants conferring resistance to rifampicin*	72.14%	rifamycin antibiotic

Antibiotic sensitivity tests showed that Lactobacillus salivarius S01 had good antibiotic sensitivity (Table 3). *L. salivarius* S01 were sensitive to some antibiotics (penicillin, ampicillin, and chloramphenicol); *L. salivarius* S01 were intermediate sensitive to tetracycline. *L. salivarius* S01 were resistant to erythromycin.

**Table 3:** Antibiotic sensitivity of *L. salivarius* S01 strains to resistance phenotypes.

	Disk content (μg)	Antibiotic sensitivity
Tetracycline	30	I
Erythromycin	15	R
Penicillin	10	S
Ampicillin	30	S
chloramphenicol	30	S

“S”: Susceptible; “I”: Intermediate; “R”: Resistant.

### Survival under simulated gastrointestinal conditions

The simulated intestinal and gastric juices tolerance of *L. salivarius* S01 at pH 2.0, 3.0, and 4.0 are shown in Table 4. The results showed that, under simulated gastric juice treatment, the survival rate of the selected strains gradually decreased at lower pH, but maintained a high survival rate (> 79.84%) at all pH conditions. Under the simulated intestinal juice treatment, the survival rate of the strain was 94.04%, indicating that the strain adapted well to intestinal conditions.

**Table 4:** Survival of *L. salivarius* S01 in simulated gastric and intestinal juices environments.

Classification	Mean of viable count (lg CFU/ mL) ± SD			Survival (%)
	Time of exposure (h)			
	0	3	4	
Simulated gastric juice (pH = 2.0)	7.54 ± 0.04	6.02 ± 0.29	——	79.84
Simulated gastric juice (pH = 3.0)		6.24 ± 0.29	——	82.76
Simulated gastric juice (pH = 4.0)		6.56 ± 0.21	——	87.00
Simulated intestinal juice	8.24 ± 0.12	——	7.75 ± 0.05	94.05

### Hepatic antioxidant enzyme activities altered By *L. salivarius* supplementation

As shown in Table 5, the levels of hepatic SOD, CAT, and POD in the experimental group fed with *L. salivarius* S01 were significantly higher than those in the control group (*p* < 0.05). Compared with the control, the SOD, CAT, and POD enzyme activity of LSM increased significantly to 25.87 ± 2.21 U/g (1.5-fold), 124.15 ± 3.91 U/g (1.8-fold), and 577.67 ± 40.22 U/g (2.0-fold), respectively (*p* < 0.05). The MDA levels were significantly decreased by 43.04% in LSM groups compared with control groups (*p* < 0.05).

**Table 5:** Effect of *L. salivarius* S01 on the activity of antioxidant enzymes (SOD, CAT, and POD) and the MDA levels in the *S. grahami* hepar.

	LSC	LSM
SOD (U/g)	17.68 ± 1.70^a^	25.87 ± 2.21^b^
CAT (U/g)	68.21 ± 4.52^a^	124.15 ± 3.91^b^
POD (U/g)	289.83 ± 31.79^a^	577.67 ± 40.22^b^
MDA (nmol/mL)	61.90 ± 2.92^a^	35.26 ± 11.71^b^

Values marked with superscripts with different letters are significantly different (P < 0.05).

### Effects of *L. salivarius* S01 On fish gut microbiota

After eliminating low-quality reads from the raw sequences of six intestinal bacterial samples of *S. grahami*, a total of 563,372 high-quality reads were obtained. These were then clustered into 572 OTUs based on the 97% 16S rRNA sequence similarity.

**Figure 4:**
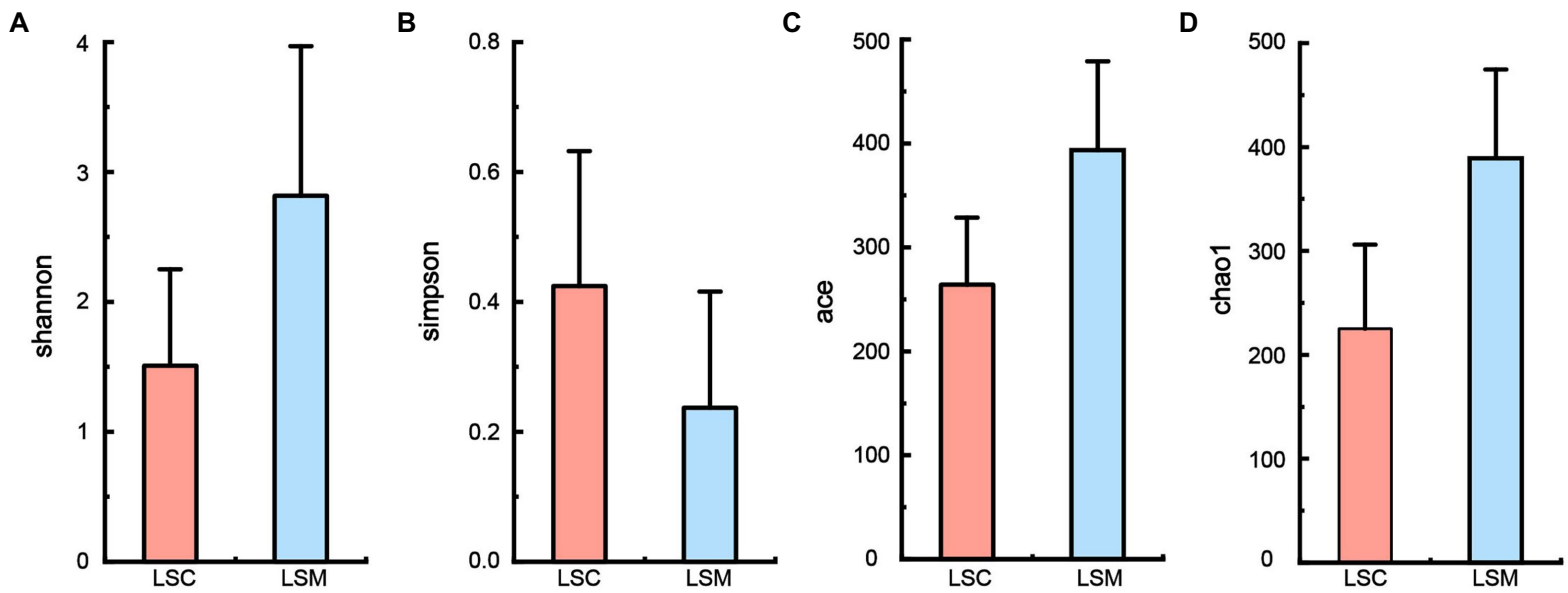
Alpha diversity of gut microbiota. **(A)** Shannon index. **(B)** Simpson index. **(C)** Ace index. **(D)** Chao1 index. *p < 0.05.

Alpha diversity indices were used to evaluate the richness and diversity of the gut microbiota in the experimental and control groups, as shown in Figure 4. Some difference was observed among the indices reflecting community richness (including Chao1 and Ace) and the indices reflecting community diversity (including Shannon and Simpson) between the two groups.

**Figure 5:**
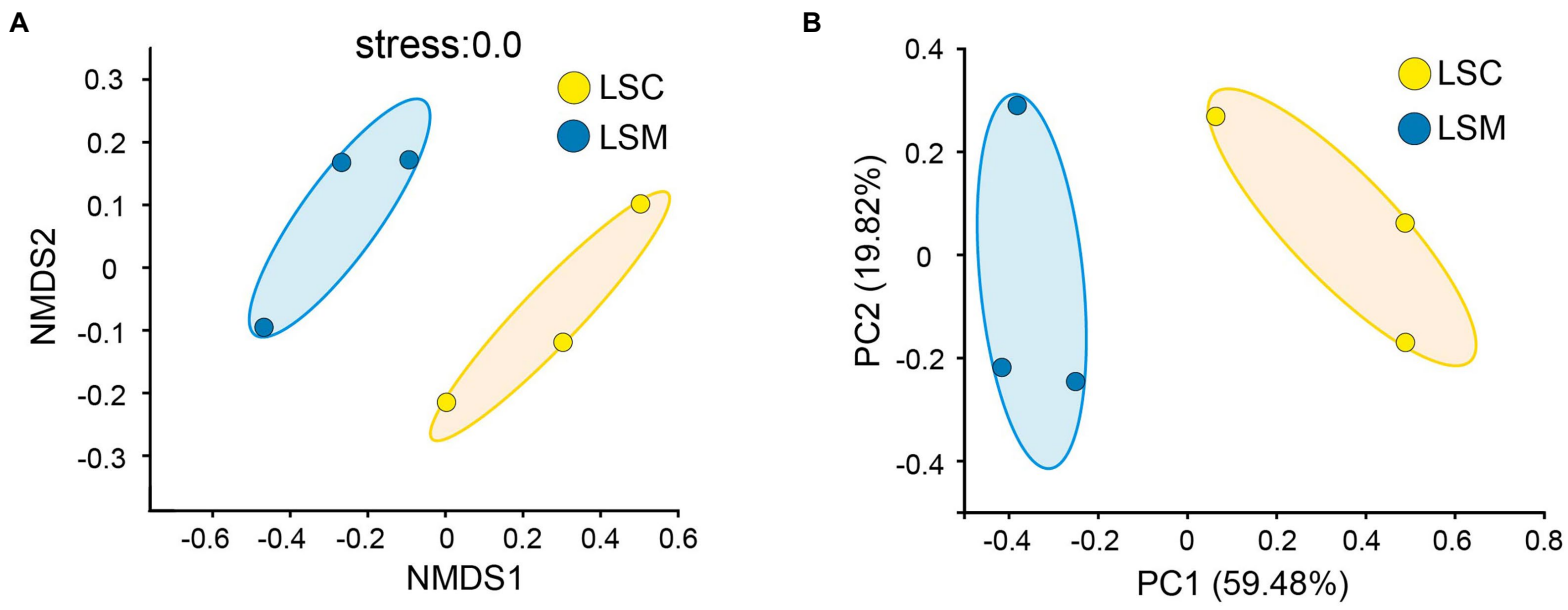
Beta diversity of gut microbiota. **(A)** Principal coordinates analysis (PCoA). **(B)** Non-metric multidimensional scaling (NMDS).

**Figure 6:**
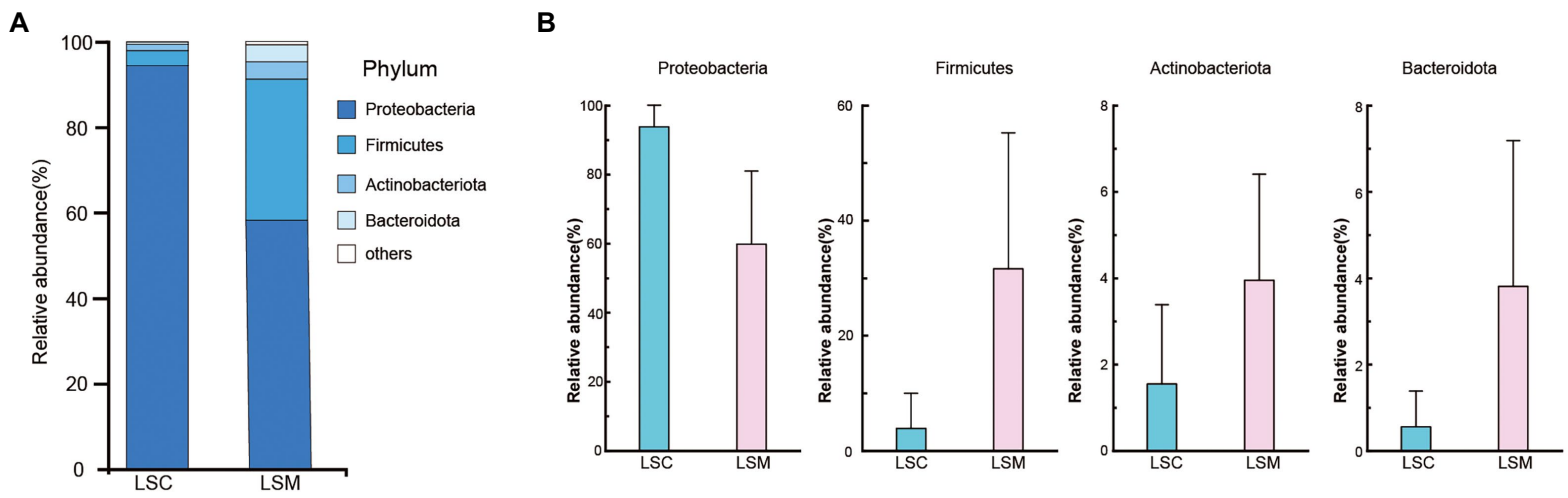
Composition of the phylum of the gut microbiota in *S. grahami*. **(A)** Relative abundance of the first five phyla. **(B)** Comparative differences at the phylum level of the gut microbiota. *p < 0.05.

The beta diversity of the samples was analyzed using principal component analysis (PCoA) and non-metric multidimensional scaling analysis (NMDS). The PCoA and NMDS results showed that the six samples were clearly divided into two clusters, consistent with the grouping (Figure 5). The reliability of the model was reflected by the stress value, equal to 0.0 in NMDS. These results demonstrated that while the bacterial supplementation did not change the richness of the gut microbiota, the community data was significantly altered compared with the control group. At the phylum level, the two groups of gut microbes were mainly composed of Proteobacteria, Firmicutes, Actinobacteriota, and Bacteroidota, among others (Figure 6). Multiple testing adjustment of the gut microbial data by Benjamini-Hochberg (BH) method. The corrected *p*-values for all the phylum level are 0.4206. In the control group, Proteobacteria was the most dominant bacterial phylum, accounting for 93.86% of all OTUs, followed by Firmicutes, Actinobacteriota, and Bacteroidota, accounting for 3.91, 1.55, and 0.56%, respectively. Compared with the control, the order of the proportion of dominant bacteria in the experimental group did not change. However, in the experimental group, the proportion of Proteobacteria decreased to 63.78% of the control group, accounting for 59.86%, and the proportion of Firmicutes increased to 8.1-fold that of the control group, accounting for 31.64%. Compared with the control group, the proportions of the Actinobacteriota and Bacteroidota phyla increased to varying degrees, accounting for 3.95 and 3.82% of the bacterial microbiota, respectively.

**Figure 7:**
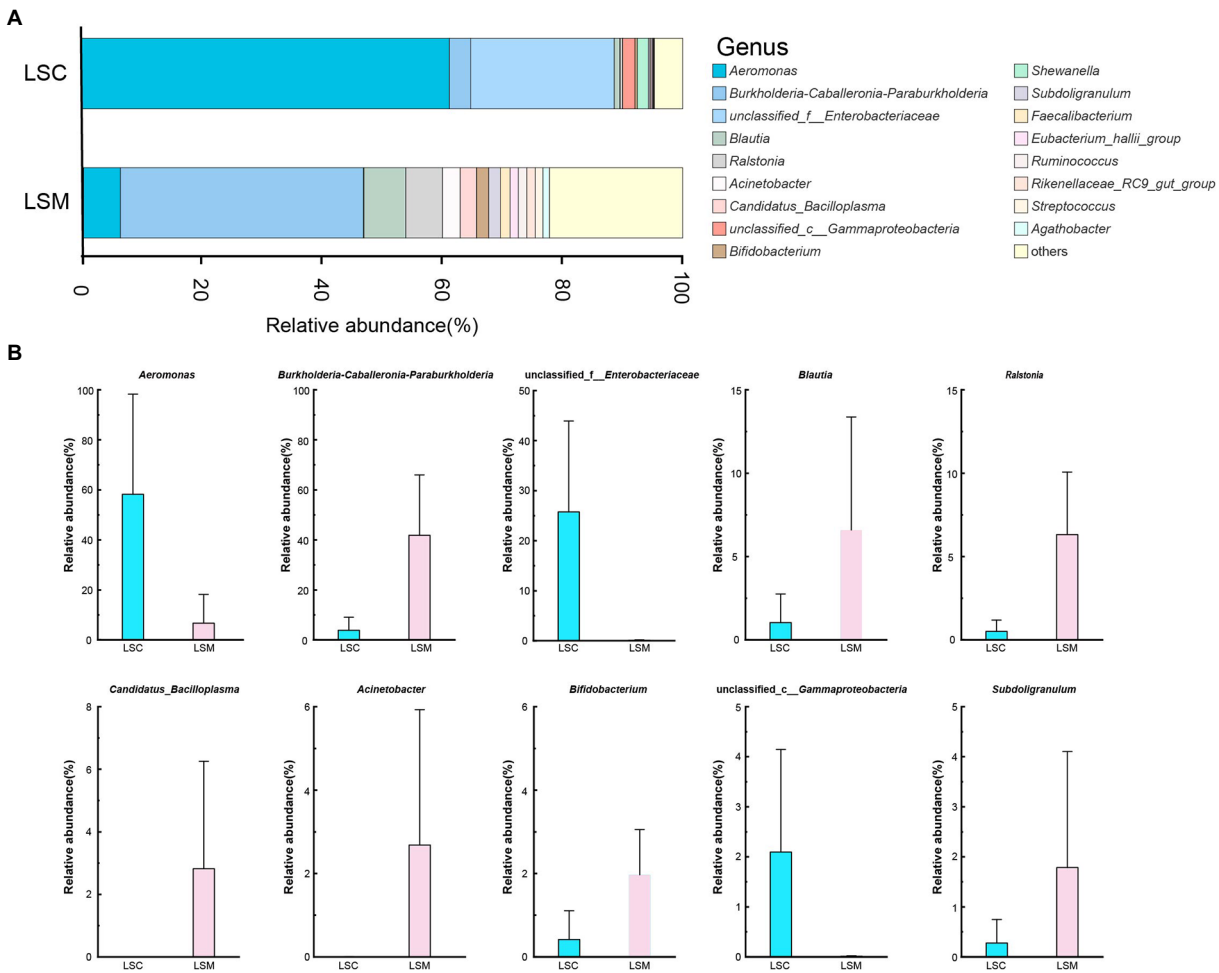
Composition of the genus of the gut microbiota in *S. grahami*. **(A)** Relative abundance of the first 17 genera. **(B)** Comparative differences at the genus level of the gut microbiota. *p < 0.05.

At the genus level, we mapped the top 10 dominant bacterial genera (Figure 7). Multiple testing adjustment of the gut microbial data by Benjamini-Hochberg (BH) method. The corrected *p*-values for the top 10 dominant bacterial genera are 0.4269. Among them, the proportion of *Aeromonas* varied between the control and experimental group. The proportion varied from 58.23 to 6.72%. The differences of the other nine genera between the control and experimental groups were not significant. *Burkholderia*-*Caballeronia*-*Paraburkholderia*, *Blautia*, *Ralstonia*, *Bifidobacterium*, and *Subdoligranulum* increased by 10.75-, 6.30-, 12.32-, 4.72-, and 6.45-fold, respectively, while *Candidatus*_*Bacilloplasma* and *Acinetobacter* increased from a negligible amount to 2.82 and 2.68%, respectively. Among them, the microbiota proportion of unclassified *Enterobacteriaceae* and *Gammaproteobacteria* decreased from 25.74 and 2.10% to a negligible amount. These results indicate that after treatment with *L. salivarius* S01, the proportion of dominant bacteria in the gut microbiota was decreased, while the overall microbiota diversity increased.

### Association of gut bacteria genus with hepatic antioxidant enzyme

**Figure 8:**
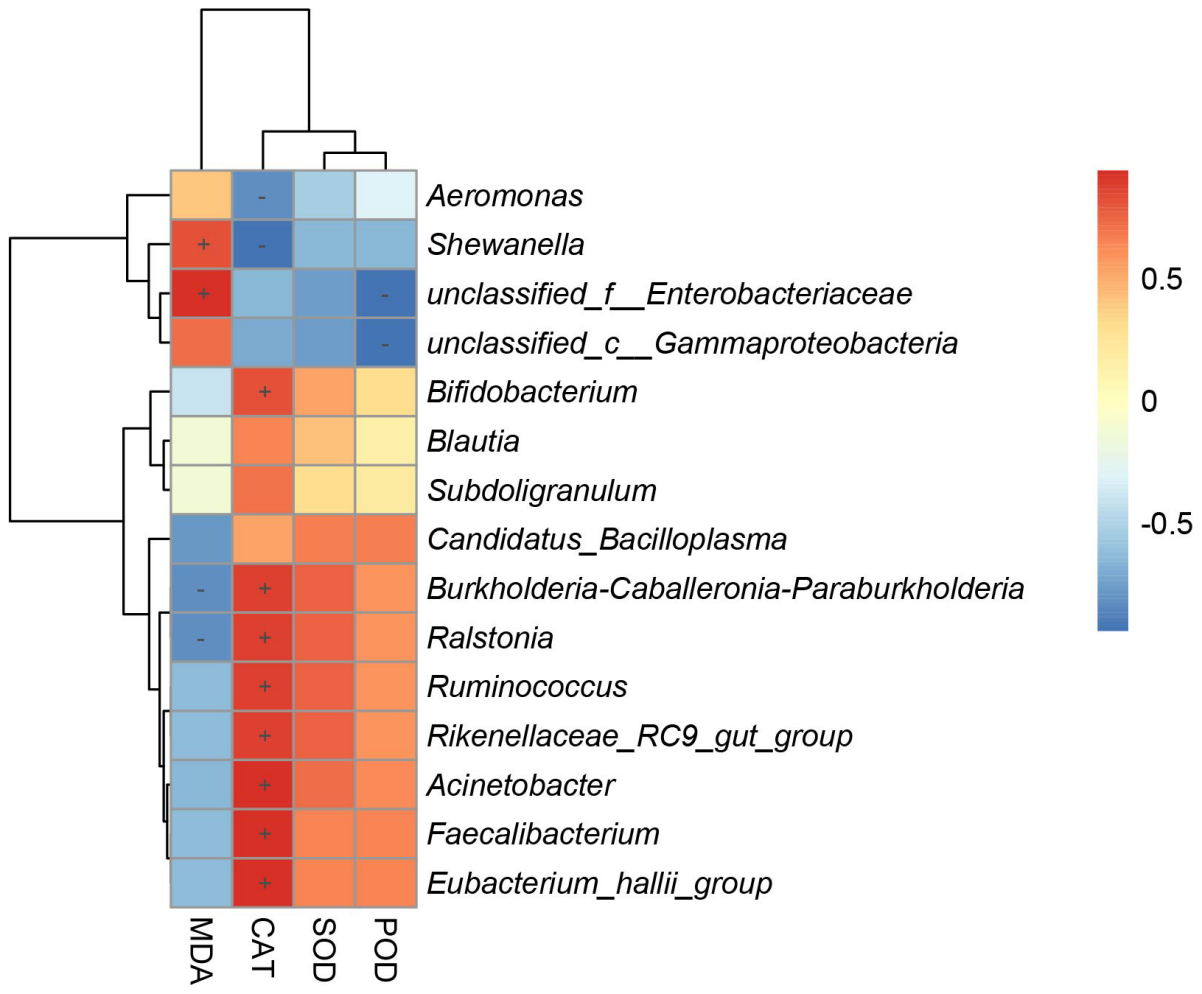
Heatmap of correlation coefficient between gut microbiota and antioxidant enzymes activity. “+” indicates a positive correlation (*p* < 0.05) and “-” indicates a negative correlation (*p* < 0.05).

To determine the gut bacteria genus involved in the antioxidant capacity observed in the host, we performed correlation analysis between gut bacterial genus and hepatic antioxidant enzyme activity (Figure 8). *Shewanella* and *unclassified_f__Enterobacteriaceae* were positively associated with the MDA contents of the hepar. *Bifidobacterium*, *Burkholderia-Caballeronia-Paraburkholderia*, *Ralstonia*, *Ruminococcus*, *Rikenellaceae_RC9_gut_group*, *Acinetobacter*, *Faecalibacterium*, and *Eubacterium_hallii_group* were positively associated with the CAT contents of the hepar. *Burkholderia-Caballeronia-Paraburkholderia* and *Ralstonia* were negatively associated with the MDA contents of the hepar. *Aeromonas* and *Shewanella* were negatively associated with the CAT contents of the hepar. *Unclassified_f__Enterobacteriaceae* and *unclassified_c__Gammaproteobacteria* were negatively associated with the POD contents of the hepar.

## Discussion

Microecological preparations can improve the immunity of the host, and other beneficial characteristics, such as a lack of drug resistance, and no toxic side effects (Mingmongkolchai and Panbangred, 2018). At present, probiotic microecological preparations are widely used in aquaculture (Gopi et al., 2022; Zhu et al., 2022). However, there is no standard for the selection of probiotics for use in microecological preparations. Host-associated probiotics isolated from the hosts may be have reportedly the most beneficial effects (Giri et al., 2014; Hao et al., 2017; Sharifuzzaman et al., 2018; Zuo et al., 2019). In this study, *L. salivarius* S01, originally isolated from the gut of *S. grahami*, was used as a feed additive in *S. grahami*. According to our previous study, a bacteriocin produced by *L. salivarius* S01 exhibited excellent inhibition effects against 12 common pathogens (both fish- and food-derived; Xin et al., 2022). This phenomenon was also confirmed by the prediction results of the antiSMASH secondary metabolite gene cluster and the BAGEL4 bacteriocin synthesis gene cluster prediction. The synthetic gene cluster of T3PKS was identified in the antiSMASH database. T3PKS expressed by the gene cluster can assist in the production of polyketides. Polyketides are a class of substances with broad antibacterial, anticancer, antioxidant, antiparasitic and anti-inflammatory activities (Bandgar et al., 2010; Mao et al., 2016; Patil et al., 2016; Vogel et al., 2008). Two bacteriocin gene clusters, Enterolysin_A and sakacin_G_skgA1, were detected in the BAGEL4 database. Enterolysin_A gene cluster encodes a cell wall degrading bacteriocin, a class III bacteriocin (Dos Santos et al., 2021; Nilsen et al., 2003). The sakacin G bacteriocin encoded by the sakacin_G_skgA1 gene cluster can lyse sensitive cells, leading to the leakage of enzymes and DNA, thereby inducing apoptosis in bacteria (Todorov et al., 2011). In addition, biochemical experiments and genomic analysis showed that Lactobacillus salivarius S01 had good antibiotic sensitivity. Therefore, *L. salivarius* S01 show great potential for use in the control of pathogens in aquaculture.

*L. salivarius* exhibits good resistance to acid and bile salt, adjusting the gut microecological balance by changing the ratio of symbiotic LAB and other bacteria, as well as reducing the gut pH (Chaves et al., 2017; Messaoudi et al., 2013). Meanwhile, *L. salivarius* has immunomodulatory, anti-inflammatory, and anti-infectious properties (Langa et al., 2012), it also can stimulate Caco-2 cells to inhibit IL-8 production, as well as promote the recovery of gut epithelial cells (Arribas et al., 2012). The tolerance of *L. salivarius* to acidic conditions plays a key role in its colonization of the intestine, thereby ensuring its probiotic potential. In this study, the results of the *in vitro* assay in a simulated gastrointestinal juices environment demonstrated that *L. salivarius* S01 was able to tolerate the extreme conditions of low pH and proteases. Meanwhile, genes associated with probiotic potential, such as environment resistance, adhesion capacity, protective repair of DNA and proteins, and antioxidant immunity, were identified in the genome. Furthermore, previous studies reported that the antioxidant capacity of animals is important because the body tends to generate reactive oxygen species (ROS) through normal cellular metabolism and in response to factors such as environmental changes and diet (Sagada et al., 2021). Hepatic antioxidant capacity (e.g., SOD, CAT, and POD) is a way of verifying the health condition and nutritional status of fish, which can regulate the balance between oxidants like ROS and antioxidants to avoid oxidative stress (Birben et al., 2012). Also, MDA is an important product of lipid peroxidation, and the level of MDA is a measure of the degree of oxidative damage (Silambarasan et al., 2019). In this study, diet supplemented with *L. salivarius* S01 significantly increased the antioxidant enzyme activity (SOD, CAT, and POD) of *S. graham*. Meanwhile, the significantly reduced malondialdehyde levels also confirmed the enhanced repair of oxidative damage by the probiotic-induced antioxidant enzyme activity. Thus, *L. salivarius* S01 was able to enter the intestine and enhance host immunity, which is necessary for probiotics to exert their beneficial effects.

The gut is home to the densest microbial populations of organisms, and plays an important role in many physiological functions, such as host metabolism and nutrition (Devillard et al., 2007). Higher gut microbial diversity provides the host with a higher tolerance to pathogens (Harrison et al., 2019). In this study, the Shannon, Chao1, and Ace indices of the gut microbiome of *S. graham* were all found to increase after feeding a diet supplemented with *L. salivarius*, indicating that *L. salivarius* can promote the gut microbial diversity and richness of the host, consistent with the alterations caused by other LAB as dietary supplements on the host gut microbiota (Ljubobratovic et al., 2017; Lukic et al., 2020). Moreover, previous studies have reported that changes in gut microbiota may be a cause of changes in host immunity (Messaoudi et al., 2013; Chaves et al., 2017; Foysal and Gupta, 2022). In this study, it is important to analyze the changes of specific microbiota in the gut microbiota. At the phylum level, we found that the abundance of the most dominant phylum Proteobacteria decreased after feeding a diet supplemented with *L. salivarius*. Elevated proportions of proteobacteria in the gut of aquatic animals increase the risk of bacterial infections caused by *Eriocheir sinensis* and *Litopenaeus vannamei* (Ding et al., 2017; Wang et al., 2019). The relative abundance of Firmicutes, Actinobacteria, and Bacteroidetes increased to different degrees after feeding *L. salivarius* compared with the control group. Firmicutes can promote the decomposition of fiber and enable the host to obtain nutrition from fibrous feed (Brulc Jennifer et al., 2009). The main role of Bacteroidetes is to degrade carbohydrates (Spence et al., 2006), and the ratio of Bacteroidetes to Firmicutes in the animal’s gut reflects the organism’s ability to absorb nutrients. In addition, the main bacteria (including *Bacillus*, *Lactobacillus*, and *Lactococcus*) in the Firmicutes phylum can convert carbohydrates into lactic acid, creating acidic environments, thereby inhibiting pathogens, and protecting the gut (Messaoudi et al., 2013; Chaves et al., 2017; Foysal and Gupta, 2022). Actinobacteria can produce a variety of compounds, including antibiotics, enzymes, enzyme inhibitors, signalling molecules, and immunomodulators (Ul-Hassan and Wellington, 2009). As such, a higher abundance of Actinobacteria in the fish gut can improve food digestion and growth performance in *S. grahami*. Furthermore, the abundance of potential pathogens *Aeromonas* in the gut tract of the *S. grahami* was found to be reduced, while the abundance of the potential beneficial microorganisms *Bifidobacterium* increased, after feeding with *L. salivarius* S01 supplements. *Aeromonas* is a common zoonotic pathogen found in fish that can cause systemic sepsis and local infections (Pereira et al., 2022). On the contrary, *Bifidobacterium* is an important indicator of good health, with nutritional, anti-tumor, and anti-aging potential, and plays an important role in regulating the balance of gut microbiota and promoting normal gut development (Di Pierro et al., 2020). Unfortunately, corrected *p*-values show a certain probability of false positive gut microbial results. In addition, correlation analysis showed that *Aeromonas* was negatively correlated with antioxidant capacity in *S. grahami*, while *Bifidobacterium* was positively correlated with antioxidant capacity. Higher levels of hepatic antioxidant enzyme activity and changes in certain gut microorganisms suggested that *L. salivarius* S01 supplements may reduce the probability of disease by enhancing the immunity of *S. grahami* against the invasion of external harmful substances. Based on genome-wide data of *L. salivarius* S01 and the bioinformatic analysis of the gut microbiota of *S. grahami*, the mechanisms by which *L. salivarius* S01 promotes host health have been elucidated. These results indicate that *L. salivarius* can be used as a potential probiotic and an antimicrobial food additive to replace chemical drugs and antibiotics in aquaculture, promoting host health and fostering the development of greener aquaculture practices. However, this study still has some drawbacks, but there are also worthwhile points to be considered.

## Conclusion

The results presented in this study demonstrated that the antibacterial activity of *L. salivarius* S01, previously isolated from the gut tract of *S. grahami* (Xin et al., 2022), may originate from the antibacterial substances produced by the bacteria, such as T3PKS, Enterolysin_A, and sakacin_G. Biological. Also, *L. salivarius* S01 showed a better antibiotic sensitivity. Function and genetics analysis related to potential probiotics showed that *L. salivarius* S01 could cope with the pressure of the natural environment, which may contribute to its colonization in the gut tract. Moreover, diet supplementation with *L. salivarius* S01 significantly altered the gut microbial diversity and hepatic antioxidant enzyme activities of *S. grahami*. Using *L. salivarius* S01 as a diet additive markedly reduced the abundance of potential pathogens and increased the abundance of potential beneficial microorganisms in the gut of *S. grahami*. Furthermore, supplementation was also found to increase the activity of antioxidant enzymes in the hepar and reduce the incidence of oxidative damage in the host. In summary, this study provides both a theoretical and experimental basis for the application of *L. salivarius* S01 as potential probiotics in aquaculture.

## Data availability statement

The datasets presented in this study can be found in online repositories. The names of the repository/repositories and accession number(s) can be found at: https://www.ncbi.nlm.nih.gov/, CP097639, SRR19351072, SRR19351073, SRR19351074, SRR19351075, SRR19351076, and SRR19351077.

## Author contributions

W-GX, X-DL, and Y-CL have carried out the culture and genomic analysis of the bacteria, they conducted the feeding of *Sinocyclocheilus grahami* and its physiological and biochemical determination, and they also wrote the paper. W-GX, Y-HJ, and M-YX contributed new reagents or analytical tools. Q-LZ, and FW reviewed and revised the manuscript. L-BL has designed the research, provided the reagents and analytical methods and written and revised the paper. All authors contributed to the article and approved the submitted version.

## Funding

This study was supported by Yunnan Major Scientific and Technological Projects (grant no. 202202AG050008).

## Conflict of interest

The authors declare that the research was conducted in the absence of any commercial or financial relationships that could be construed as a potential conflict of interest.

## Publisher’s note

All claims expressed in this article are solely those of the authors and do not necessarily represent those of their affiliated organizations, or those of the publisher, the editors and the reviewers. Any product that may be evaluated in this article, or claim that may be made by its manufacturer, is not guaranteed or endorsed by the publisher.

## Supplementary material

The Supplementary material for this article can be found online at: https://www.frontiersin.org/articles/10.3389/fmicb.2022.1014970/full#supplementary-material

SUPPLEMENTARY FIGURE S1 Histogram of Gene Ontology (GO) enrichment analysis results for the *L. salivarius* S01 genome.

SUPPLEMENTARY FIGURE S2 Histogram of Kyoto Encyclopedia of Genes and Genomes (KEGG) enrichment analysis results for the *L. salivarius* S01 genome.
