# Supplementary figures and images for: Whole genome analysis of host-associated lactobacillus salivarius and the effects on hepatic antioxidant enzymes and gut microorganisms of Sinocyclocheilus grahami
Source: Front Microbiol. 2022 Oct 27;13:1014970. doi: 10.3389/fmicb.2022.1014970 (PMC9648147; doi:10.3389/fmicb.2022.1014970)

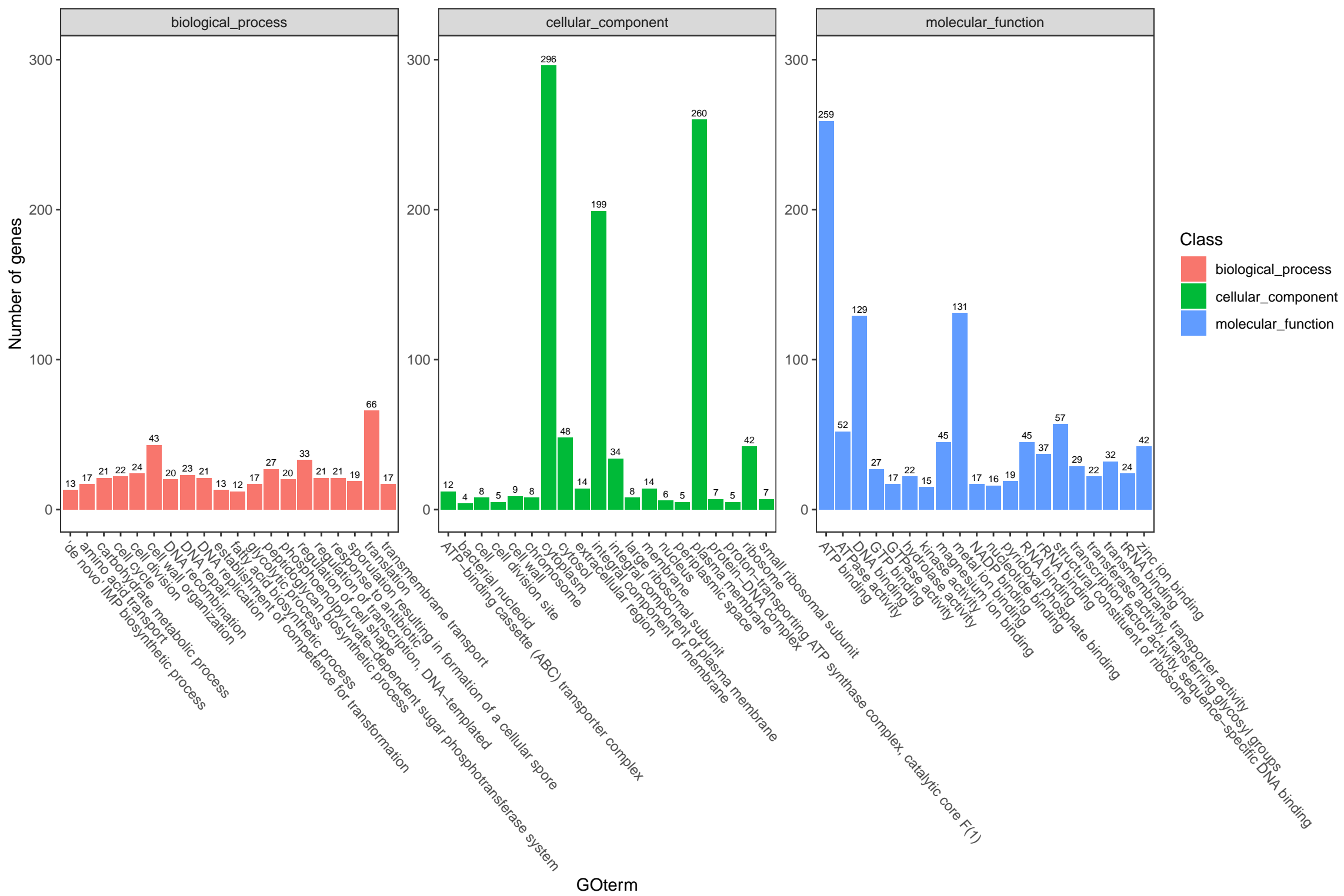

Supplement: SUPPLEMENTARY FIGURE S1 — Histogram of Gene Ontology (GO) enrichment analysis results for the L. salivarius S01 genome. [file Data_Sheet_1.PDF]

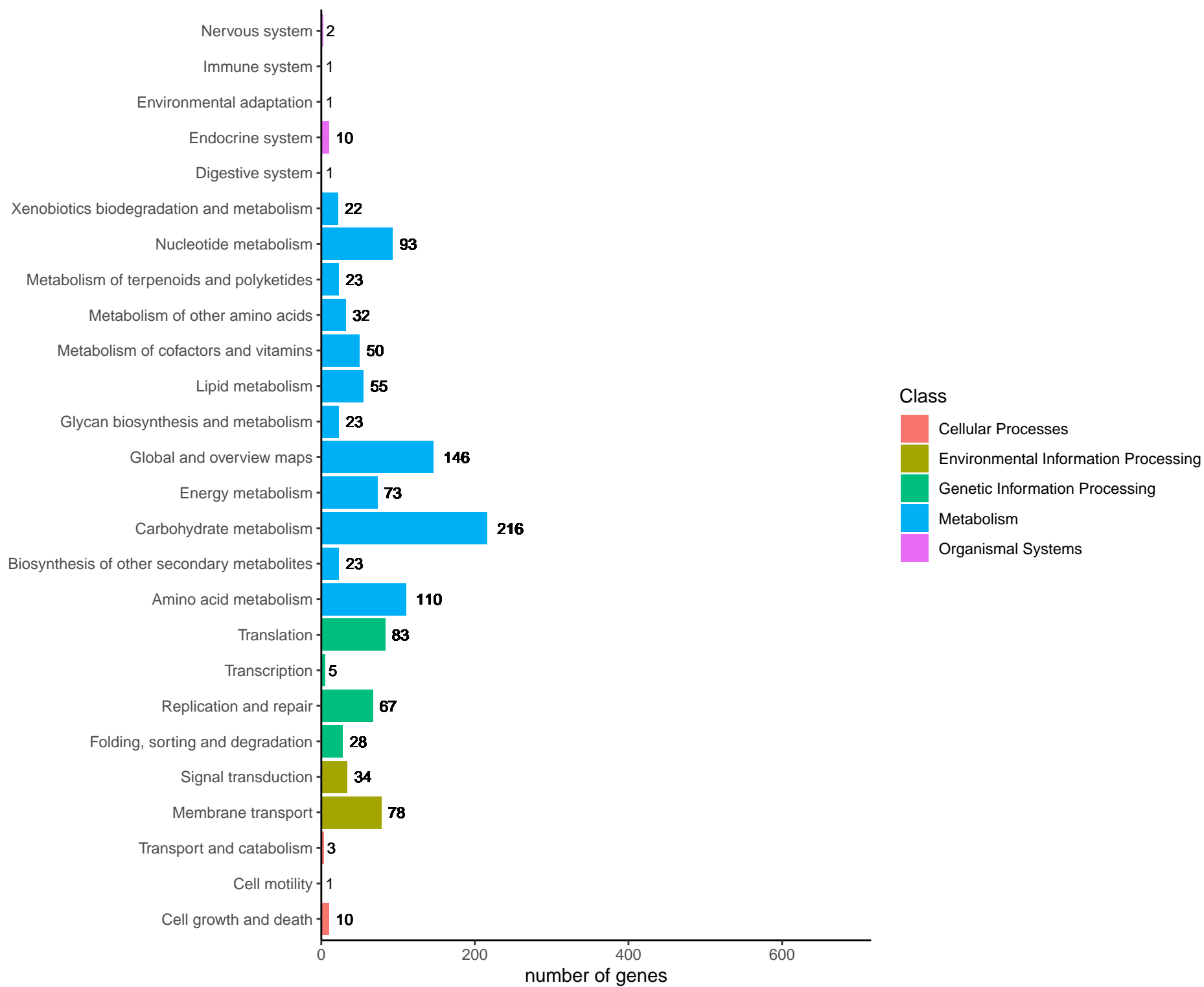

Supplement: SUPPLEMENTARY FIGURE S2 — Histogram of Kyoto Encyclopedia of Genes and Genomes (KEGG) enrichment analysis results for the L. salivarius S01 genome. [file Data_Sheet_2.PDF]
